# Autophagy Induction by *Scutellaria* Flavones in Cancer: Recent Advances

**DOI:** 10.3390/ph16020302

**Published:** 2023-02-15

**Authors:** Hardeep Singh Tuli, Sakshi Bhushan, Ajay Kumar, Poonam Aggarwal, Katrin Sak, Seema Ramniwas, Kanupriya Vashishth, Tapan Behl, Rashmi Rana, Shafiul Haque, Miguel A. Prieto

**Affiliations:** 1 Department of Biotechnology, Maharishi Markandeshwar Engineering College, Maharishi Markandeshwar (University), Mullana, Ambala 133207, India; 2 Department of Botany, Central University of Jammu, Samba 181143, India; 3 Punjab Biotechnology Incubator (P.B.T.I.), Phase VIII, Mohali 160071, India; 4 The Basic Research Laboratory, Center for Cancer Research, National Institutes of Health, Frederick, MD 20892, USA; 5 NGO Praeventio, 50407 Tartu, Estonia; 6 University Centre for Research and Development, University Institute of Pharmaceutical Sciences, Chandigarh University, Gharuan, Mohali 140413, India; 7 Advance Cardiac Centre Department of Cardiology, Post Graduate Institute of Medical Education and Rsearch (P.G.I.M.E.R.), Chandigarh 160012, India; 8 Department of Pharmacology, School of Health Sciences & Technology (SoHST), University of Petroleum and Energy Studies, Bidholi, Dehradun 248007, India; 9 Department of Research, Sir Ganga Ram Hospital, New Delhi 122016, India; 10 Research and Scientific Studies Unit, College of Nursing and Allied Health Sciences, Jazan University, Jazan 45142, Saudi Arabia; 11 Gilbert and Rose-Marie Chagoury School of Medicine, Lebanese American University, Beirut P.O. Box 13-5053, Lebanon; 12 Centre of Medical and Bio-Allied Health Sciences Research, Ajman University, Ajman P.O. Box 346, United Arab Emirates; 13 Nutrition and Bromatology Group, Faculty of Food Science and Technology, University of Vigo, Ourense Campus, E32004 Ourense, Spain

**Keywords:** *Scutellaria* flavones, chemistry, autophagy, cancer

## Abstract

In parallel with a steady rise in cancer incidence worldwide, the scientific community is increasingly focused on finding novel, safer and more efficient modalities for managing this disease. Over the past decades, natural products have been described as a significant source of new structural leads for novel drug candidates. *Scutellaria* root is one of the most studied natural products because of its anticancer potential. Besides just describing the cytotoxic properties of plant constituents, their molecular mechanisms of action in different cancer types are equally important. Therefore, this review article focuses on the role of the *Scutellaria* flavones wogonin, baicalein, baicalin, scutellarein and scutellarin in regulating the autophagic machinery in diverse cancer models, highlighting these molecules as potential lead compounds for the fight against malignant neoplasms. The knowledge that autophagy can function as a dual-edged sword, acting in both a pro- and antitumorigenic manner, further complicates the issue, revealing an amazing property of flavonoids that behave either as anti- or proautophagic agents.

## 1. Introduction

In ethnomedicine, plants have been used for the treatment of various health problems for centuries [1]. Even today, more than 80% of the world’s population relies on herbal medicines for their primary health care [2]. Considering malignant disorders, a more systematic approach to herbal medicines started with the launch of a large-scale screening program by the U.S. National Cancer Institute in 1960, leading to the isolation and characterization of several novel anticancer agents and inspiring researchers all over the world to investigate the infinite molecular abundance of the plant kingdom [3,4,5].

**Figure 1:**
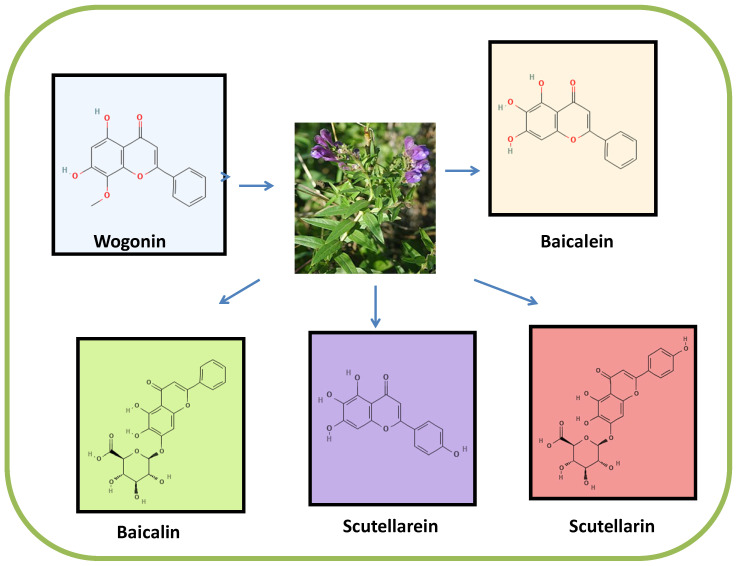
The most important flavone constituents of *Scutellaria* plants.

*Scutellaria*, a genus of flowering plants from the family Lamiaceae that is usually known as skullcaps, is one of the most attractive herbal resources of polyphenolic compounds with anticancer properties. Indeed, several important phytochemicals from the class of flavonoids have been isolated from the roots of *Scutellariae* plants, including wogonin, wogonoside, baicalein, baicalin, scutellarein and scutellarin (Figure 1) [6,7]. The various anticancer mechanisms of these flavones have been described in different cancer cell cultures and xenografted animal preclinical models, demonstrating the potential of these compounds to exert anti-inflammatory, cell death-inducing, antimetastatic and antiangiogenic properties in different cancer types [8,9,10,11]. Furthermore, among several other mechanisms, *Scutellaria* flavones have been shown to suppress malignant growth through the modulation of autophagy [12,13]. Moreover, depending on various factors, such as the cancer cell type, activated cellular signaling network and molecular characteristics of the isolated compounds, Scutellaria-derived flavonoids can exert antitumor activity [13,14] by inducing autophagic processes [12,15]. The present review focused on the potential of Scutellaria flavones to regulate the autophagic machinery in diverse cancer models. Flavones are the major group of naturally occurring phytochemical substances that have attracted substantial attention due to their chemotherapeutic and/or pharmacological properties [16]. *Scutellaria* flavones are generally derived from the *Scutellaria baicalensis* Georgi that show many pharmacological properties, such as antioxidation, antiviral, hepatoprotective, etc. [17], and *Scutellaria* flavones have shown apoptosis-inducing properties in many cancer cell lines [18].

Numerous aspects of Scutellaria flavones related to autophagy death in cancer cells are covered in this review. The authors surveyed the literature, and few reviews showed the autophagy-targeting pathways in the cancer models using Scutellaria flavones. That is why the authors highlighted the autophagy-signaling pathways modulated by *Scutellaria* flavones.

In this review article, the chemical features of the flavone-type compounds of the *Scutellaria* root are described by analyzing their autophagy-modulating abilities in diverse malignant models to better understand the complex anticancer mechanisms of these agents in different malignant systems. In addition, the absorption and metabolic profiles of these important molecules within in vivo systems are discussed to present, in addition to attractive anticancer effects, the potential bottlenecks for initiating clinical trials in the future.

## 2. Sources and Chemistry of *Scutellaria* Flavones (Wogonin, Baicalein, Baicalin, Scutellarein and Scutellarin)

### 2.1. Wogonin

Plants possess a plethora of secondary metabolites, including polyphenolic flavonoids. They are naturally distributed in the pigments, flowers, stems, seeds, fruits, vegetables, spices and flowers [19]. Humans are exposed to these phytonutrients of therapeutic importance through various sources. Among the various flavonoids, 5, 7-dihydroxy-8-methoxy flavone, also known as wogonin, is an important derivative of flavones. In the year 1930, wogonin was first isolated from *Scutellaria baicalensis* (Lamiaceae), which is a widely distributed family in North America, Russia and East Asia [20]. Wogonin naturally occurs in the whole herb of *S. baicalensis,* including the roots [21], and in various plants, such as *Andrographis paniculata* Burm.f (leaves), *Anodendron affine* (Hook. & Arn.) Druce (stem) [22,23] and *Tetracera indica* L. (leaves and stem) [20]. It can be synthesized/obtained via extraction, a chemical process involving cyclization of 1,3- diaryl-diketone, or with Wessely–Moser rearrangement [24]. It has been observed that the chemical synthesis of this compound is a convenient method to obtain it in large quantities, which involves using trimethoxyphenol as the starting material [25].

Wogonin is a monoflavonoid with a molecular weight of 284.267 g/mol and solubility in organic solvents, such as ethanol, dimethylformamide (D.M.F.) and dimethyl sulfoxide (DMSO). Interestingly, many of the therapeutic properties of the *Scutellaria* species viz., including antioxidant (Gao et al., 1999), antiviral [26], anti-inflammatory [27], antiproliferative [6] and multi-drug resistant [28], can be attributed to wogonin along with other phytoconstituents (Table 1).

### 2.2. Baicalein

Baicalein (5,6,7-trihydroxyflavone) is one of the naturally occurring flavones, an important class of flavonoids, originally isolated and extracted from *Scutellaria baicalensis* and *Scutellaria lateriflora* [29,30]. It has also been reported in *Oroxylum indicum* and *Thyme*. Baicalein is a structural derivative of 1-benzopyran phenylpropanoid and a functional parent of “Bicalin” [31]. Chemically, it is an aglycone of “baicalin” having a hydroxyl group at the C-5, C-6 and C-7 positions. Its molecular structure shows the presence of di-orthohydroxyl as a functional group associated with the ring –A. Interestingly, this phytocompound is a key ingredient of Sho–Saiko To, an herbal formula of Chinese origin, and is also related to “Kempo medicine“ of Japanese origin. Baicalein acts as a “positive allosteric modulator” of the GABAA receptor at the benzodiazepine/non-benzodiazepine site (Table 1) [32]. It also exhibits selectivity for the α2 and α3 subunits in the GABAA receptors due to its structural feature, which is responsible for its anxiolytic property [29].

### 2.3. Baicalin

One of the most important phytoconstituents of *Scutellaria baicalensis* is baicalin, also known as 5,6-dihydroxy-4-oxygen-2-phenyl-4H-1-benzopyran-7-β-D-glucopyranose acid [33]. It is the critical constituent of the *Scutellaria* species and is crucial to its pharmacological and therapeutic functions [34]. “Baicalin” is present in abundant quantities in the stem and leaves of the *Scutellaria* species of the Lamiaceae family. Interestingly, Baicalin has been utilized for centuries as a traditional Chinese herbal medicine for curing psoriasis, inflammation, hypertension, cardiovascular diseases, etc. [35]. Other plant species in which it is present and isolated are *S. lateriflora*, *S. galericulata* and *Thalictrum baicalense* [36]. Baicalin has also been reported to have been purified from *Radix scutellariae* via the process of uridine diphosphate glucuronidation. Moreover, it is a “flavone glycoside” and, upon further hydrolysis, is converted to an aglycone “baicalein” [37,38]. It is also called glycosyloxyflavone, which represents the “7-O-glucuronide” of baicalein and the conjugated acid of a baicalin(1-). The various bioprotective roles of baicalin include its ability to act as a neuroprotective, cardioprotective, antioxidant, antiatherosclerotic, antibacterial and anticoronaviral agent [39] (Table 1).

### 2.4. Scutellarein

Scutellarein is a naturally occurring flavone, effectively isolated from *Scutellaria baicalensis* Georgi along with *Scutellaria lateriflora*, *Scoparia dulcis*, *Artemisia douglasiana* and *Asplenium belangeri* (Fern) [40]. It is chemically designated as 6-hydroxyapigenin or 4’,5,6,7-tetrahydroxyflavanone and shows the presence of hydroxy groups at the C-4’, -5, -6 and -7 positions. Moreover, it is functionally related to apigenin [41]. Recent research has focused on scutellarein owing to its interesting bioprotective properties, including anticancer, antiproliferative and antioxidant activities [42] (Table 1). Interestingly, being a flavone, its structure is based upon the backbone of -phenylchromen-4-one (2-phenyl-1-benzopyran-4-one) [43]. Therefore, it is known as a lipid molecule. The solubility of scutellarein is observed to be less in water and shows weak acidic properties. Furthermore, it is efficiently produced from “apigenin” [36]. Apigenin, 4’,5,7-trihydroxy-flavone, is a monomeric flavonoid found in the daily diet [44]. Apigenin has gained attention among researchers partly due to being nonmutagenic and of low toxicity compared to related flavonoids [45]. Scutellarein was used to prepare other compounds, such as scutellarin, 4’,6-dihydroxy-5,7-dimethoxyflavone and 6-hydroxy-4’,5,7-trimethoxyflavone.

### 2.5. Scutellarin

Scutellarin is a flavonoid frequently derived from the genus *Scutellaria* and *Erigeron* from Asteraceae [32]. It is widely used in the preparation of herbal medicine due to its numerous pharmacological properties [38] (Table 1). Chemically, it is a glucuronide conjugate of 5,6,7,4′-tetrahydroxyflavone at the “7-O position”. Scutellarin is a monosaccharide derivative whose chemical analogs share a common skeleton of the flavonoid/s. Scutellarin, a phenolic natural compound, is extensively used to treat various ailments, viz., liver diseases [46,47], cerebrovascular diseases [48] and hyperlipidemia [49,50]. Recent reports have indicated that over 10 million Chinese patients depend on scutellarin and associated drugs, indicating its therapeutic potency [51].

## 3. Absorption and Metabolism of *Scutellaria* Flavones

Since flavonoids are important phytoconstituents of plants, it is essential for their distribution, absorption and metabolism in plasma and tissue after ingestion. Therefore, this approach helps determine the concentrations and forms of such compounds in plasma and tissue. In addition, most flavonoids tend to bind sugars as b-glycosides, which further determine their absorption directly from the small intestine or through the colon [42]. Interestingly, before absorption, the dietary flavonoids must be released by chewing or digestion via digestive juices (gastrointestinal tract) followed by the action of microorganisms in the colon. Overall, it depends upon the physio-chemical properties of the flavonoids, including the molecular size, configuration, solubility, lipophilicity, etc.

After being absorbed by the small intestine, flavonoids are often conjugated with glucuronic acid. Due to the absence of free flavonoid aglycones in the plasma or urine, efficient absorption is achieved. When aglycones and glucosides are taken together, isoflavones are found to have the best bioavailability of all the subclasses of flavonoids. In contrast, anthocyanins have the lowest bioavailability and are quickly absorbed. Moreover, the elimination half-lives of different flavonoid subclasses vary from flavonols [53,54].

Importantly, the metabolism of flavonoids involves two key compartments: the small intestine, liver and kidneys, which represent the first compartment, and the colon, which constitutes the second compartment [55]. The flavonoids absorbed (followed by bile secretion) and unabsorbed (from the small intestine) reach the colon, which is a crucial step in the overall metabolism of these constituents. In the first section/compartment, the flavonoids and their colonic metabolites are biotransformed through enzymatic action, as evident from the conjugation of polar hydroxyl groups with glucuronic acid [53]. Additionally, the process of O-methylation plays a critical role in the inactivation of the catechol moiety if present. Additionally, recent research has revealed that glycosides are deglycosylated in the intestine [56]. As a result, after ingesting flavonoids, the conjugated metabolites of these compounds can be identified in the plasma. Most flavonoids prefer/undergo sulfation, methylation and glucuronidation in the small intestine and liver. Fascinatingly, flavonoids are found to have quick urine and biliary excretion but low intestinal absorption [57]. Keeping this in mind, the present review highlighted the various pharmacokinetic studies involving flavonoids from *Scutellaria* that are documented in a tabular form (Table 2).

## 4. Mechanistic Role of Autophagic Death in Cancer

According to the type of tumor and stage of oncogenic development, autophagy can –play a dual-edged sword role by acting as both pro- and antitumorigenic (sikder et al.; 2022 [66]). However, the role of autophagy in cancer is controversial and complicated.

### 4.1. Antitumorigenic

**Figure 2:**
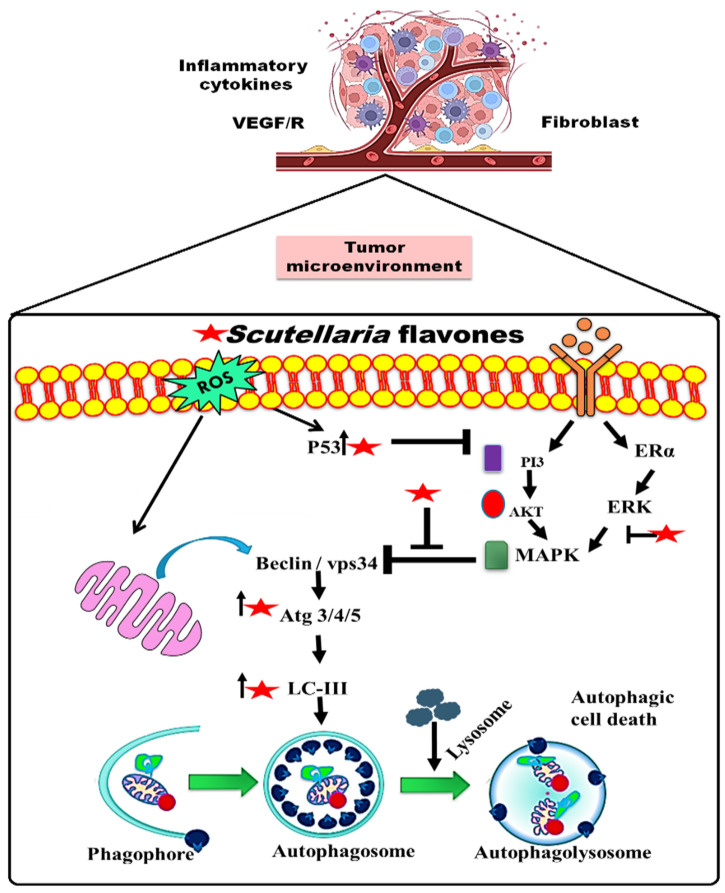
Schematic diagram showing the role of *Scutellaria* flavonoes in the tumor environment.

During the early stages of cancer, autophagy plays an important role in removing aggregated misfolded proteins, damaged mitochondria and other cellular organelles, thereby protecting the cells from further genomic instability [77,78,79]. In addition, autophagy may initiate the cell death machinery in the cancer cells with impaired apoptotic cell death. Growing solid tumors that have an autophagy defect at early stages is reported to show chemotherapeutic drug resistance. Therefore, the induction of autophagy may increase cell death either by mitophagy or by apoptosis. In vitro studies have shown that EB1089 (an analogue of vitamin D) and arsenic trioxide can promote autophagy-induced apoptotic cell death [80,81]. Treatment with specific chemotherapeutic agents, such as dexamethasone, etoposide and fenretinide, induces autophagy-mediated cell death in vitro [82,83,84]. These autophagy-inducing drugs act by blocking the activity of mTOR, possibly by activating AMPK. Rapamycin is reported to stabilize the raptor–mTOR complex by binding with FKBP12 and, thus, suppress mTOR activity, resulting in autophagy induction [85]. Similar findings are also reported in neuroblastoma cells, hepatocellular carcinoma (HCC) and murine sarcoma, where autophagy-mediated cell-cycle arrest suppressed tumor growth [86,87,88]. Antioncogenes connected to autophagy, such as p53 and Beclin-1, as well as phosphatase and the tensin homolog on chromosome 10 (PTEN) (Figure 2) play important roles in controlling carcinogenesis. For instance, the growth of autophagosomes depends on Beclin-1 [89,90]. PTEN initiates autophagy and inhibits the PI3K/Akt/mTOR pathway, controlling the cell cycle and cell proliferation [90,91]. By activating TSC1/2 and AMP-activated protein kinase, p53 regulates autophagy and inhibits mTOR [90].

### 4.2. Protumorigenic

Autophagy helps tumor cells survive by providing higher amounts of nutrients and oxygen during metabolic and hypoxic stress [92,93,94]. Moreover, Beclin1-UV irradiation-resistance-associated gene (UVRAG) core complex-induced autophagy confers resistance to radiation-induced DNA double-strand breaks (DSBs) in tumor cells [95,96]. Paglin et al. first observed the formation of acidic vacuoles in neoplastic cells when exposed to IR that helps tumor cells survive [95]. Interestingly, the role of autophagy in tumor cell survival is not only restricted to the tumor vicinity, while hypoxia inducible factor-1α (HIF-1α) induced autophagy also plays an important role in metastasis [97]. Autophagy can induce resistance to various cancer therapies, leading to chemo resistance and cancer cell survival [98,99,100]. Therefore, inhibition of autophagy with various pharmacological inhibitors, such as 3-methyladenine (3-MA, a PI3K III inhibitor), Bafilomycin A (inhibitor of vacuolar-type H+-ATPase), chloroquine (CQ) or hydroxychloroquine (HCQ) (impair autophagosome fusion with lysosomes), or with genetic deletion of autophagy-related genes, such as ATG5, ATG6 and ATG7, enhance the effect of various anticancer therapies [101,102,103,104,105].

Numerous studies demonstrated that the activation of tyrosine kinases (T.K.s) and receptor tyrosine kinases (RTKs) that include EGFR, PDGFR, RAF and VEGFR modifies autophagy by triggering various signaling pathways, such as PI3K/AKT/mTORC1 and RAS/MAPK [106,107]. While autophagy functions in the wake of RTK pathways, there is also a reciprocal relationship between autophagy and RTK signaling. RTKs can restrict autophagy through mTORC1, while autophagy can also favorably influence RTK signaling through activation of mTORC2 [108].

Effective antitumor therapies have been created using tyrosine kinase inhibitors (T.K.I.s). The catalytic domain of tyrosine kinase is where T.K.I.s and ATP fight for a binding site (T.K.s) [109]. The F.D.A. has authorized numerous T.K.I. drugs, including imatinib (PDGFR, ABL kinase), mesylate, erlotinib and gefitinib (EGFR TKIs), lapatinib (EGFR/HER2), Sunitinib (VEGF, PDGFR) and sorafenib (VEGFR kinase, RAF, PDGFR), for use in human clinical trials [110,111].

As autophagy has both pro- and anticancer effects, proper tumor stage and tumor type need to be considered accurately to answer the question whether to induce or suppress autophagy. Many clinical trials targeting autophagy are now being conducted to increase the effectiveness of medicines that modulate autophagy to treat cancer.

## 5. Regulation of Autophagy by *Scutellaria* Flavones

### 5.1. Wogonin and Autophagy Induction

Wogonin is a flavonoid obtained from *Scutellaria baicalensis*; studies have exhibited the prominent role of wogonin in inhibiting malignant tumor growth via inducing apoptosis and regulating autophagy [112,113]. Autophagy plays an important role in disassembling the unnecessary or dysfunctional components of the cell. It acts as a regulatory and destructive mechanism [12,114,115]. Studies show that autophagy contributes to tumor suppression via different inhibiting mechanisms [114,115,116]. Studies show the role of wogonin in strengthening the efficacy of anticancer drugs and offering lower toxicity when used in combination. Different studies highlight the role of Unc-51-like autophagy-activating kinase 1 (ULK1), protein kinase B (AKT), eukaryotic initiation factor 4E-binding protein 1 (4E-BP1), mammalian target of rapamycin (mTOR) and cylindromatosis (CYLD) in mediating autophagy. Investigations demonstrate the role of wogonin in upregulating and downregulating the expression of these molecules involved in autophagy in human pancreatic cancer cells (HPCCs) [116,117,118,119]. It was shown that wogonin regulated autophagy via ULK1, as it is the key regulator of autophagy. It coordinates the earliest phases of autophagosome production when combined with oxaliplatin to stimulate autophagy in human gastric (BGC-823) cells. The study showed that wogonin reduced the phosphorylation necessary for mitochondrial homeostasis and cell survival during starvation, either alone or in combination with oxaliplatin. The study’s findings showed that combining oxaliplatin and wogonin promotes excessive autophagy by excessive nitrosative stress, amplifying oxaliplatin-induced cell death. Studies report that wogonin promotes reactive oxygen species (ROS) formation in cancer cells, and the overgeneration of ROS in human gastric cancer cells is linked with autophagy [120]. Zhang et al. reported that wogonin and icotinib, when used in combination, activated autophagy and increased the rate of apoptosis in lung cancer cells (NCI-H1975), suggesting the role of autophagy in apoptosis. The study demonstrated that the combined effect of wogonin and icotinib increased the phosphorylation level of mTORC1, a classical regulator of autophagy, thereby promoting autophagy [121]. Li et al. also reported that wogonin significantly downregulated mTOR and upregulated ULK1, AKT, 4E-BP1 and CYLD expressions in human pancreatic cancer cells (HPCCs), throwing light on the role of wogonin-induced autophagy activators [119].

Further, the study demonstrated that wogonin activated Beclin-1/PI3K, enhancing ROS generation and ROS-mediated autophagy [119]. According to studies, wogonin controls the Bax/Bcl-2 and c-myc signaling pathway to cause apoptosis [122]. Many health benefits, including anticancer properties, are attributed to wogonin. Many in vitro studies emphasize the role of wogonin in the regulation of autophagy in cancer, and translating the beneficial properties of wogonin from bench side to bedside in cancer management could prove to be a useful strategy to exploit the therapeutic potential of wogonin (Table 2).

### 5.2. Baicalein and Baicalin Induce Autophagy

*Scutellaria* root contains the flavonoid derivative chemicals baicalein and baicalin. Both have potent antioxidant and anti-inflammatory activities and are often nontoxic to human cells [123,124]. Moreover, many studies have indicated their antitumor and antimetastatic roles in various cancer types [125,126,127]. Different mitochondrial/endoplasmic reticulum pathways mediated by reactive oxygen species and inhibition of AKT are responsible (Table 2) for the transmission of these effects [128,129,130,131]. For instance, Aryal et al. confirmed that baicalein induced autophagy in cancer cells rather than apoptosis. They showed that the baicalein-induced cell death was completely reversed when autophagy was suppressed by inhibiting the expression levels of molecules, such as Beclin-1, vacuolar protein sorting 34 (Vps34), autophagy-related (Atg)5 and Atg7, but not by a caspase inhibitor. Their findings suggested that baicalein enhanced the autophagic flow in addition to autophagosome formation. Moreover, their data suggested that the anticancer autophagic role of baicalein is mediated through upregulation of the AMPK/ULK1 pathway and downregulation of mTOR/Raptor complex 1 expression [132]. A similar study in ovarian cancer cells confirmed the role of baicalein in inducing Beclin-1-mediated autophagy. They also showed that baicalein-mediated autophagy is associated with extracellular signal-regulated kinase (ERK, Thr202/Thr204) and AKT (Ser473) phosphorylation [133]. Wang Z et al. reported that only baicalein, among four different flavonoids, showed an antitumor effect on hepatic cellular carcinoma (H.C.C.). They further showed the role of E.R. stress in inducing apoptosis and protective autophagy triggered by baicalein [134]. In a Lewis lung cancer (L.L.C.) xenograft model, a recent study demonstrated the important function of the A.M.P.K./mitochondrial fission pathway in regulating baicalein-induced apoptosis and autophagy. Even mitochondrial abnormalities were restored by reducing A.M.P.K. activation [135]. Together, these two studies indicate that the mitochondrial/E.R. axis plays a potent role in the baicalein-induced antitumor effect. Another intriguing study discovered that baicalein plays a dose- and time-dependent effect in triggering autophagy in undifferentiated follicular thyroid cancer cells (F.R.O.). Beclin-1, Atg5, p62 and Atg12 expression levels were significantly upregulated, and the ERK and PI3K/Akt pathways were inhibited. [136]. Baicalein was even shown to induce cell cycle arrest in cervical cancer cells by reducing the Cyclin D1 protein concentration and modulating the AKT/mTOR signaling pathway [137]. Aberrant activation of AKT serine/threonine protein kinase modulates autophagy in gastric cancer cells [138]. The synergistic role of baicalein along with cisplatin (DDP) has been shown to have promising antitumor effects on even DDP-resistant SGC-7901/DDP gastric cancer. The combined impact was more potent than DDP or baicalein acting separately. By triggering apoptosis and autophagy via the Akt/mTOR and Nrf2/Keap 1 pathways, baicalein increased the DDP sensitivity of SGC-7901/DDP gastric cancer cells [139]. In addition, baicalin also upregulated intercellular Ca^2+^ and ROS, associated with downregulating the PI3K/Akt/mTOR, ERK1/2 and β-catenin signaling pathways. Chelation of free Ca^2+^ with BAPTA-AM also downregulated apoptosis induction and ROS accumulation. Consequently, baicalin may be observed as a viable contender for the diagnosis of osteosarcoma [140].

### 5.3. Scutellarein and Scutellarin Induce Autophagy

Scutellarin and scutellarein are the natural oxyflavonoids obtained from *Erigeron breviscapus*; they are naturally glycosylated, perform a wide range of functions and have a variety of medicinal properties [13,141,142]. The therapeutic properties are as follows: vasodilating, acting as an anticoagulant and being tumor suppressive. Different cumulative reports show the repressive effects of scutellarin and scutellarein on cancers, such as liver, lung, colon, lymphoma, etc. Studies report that cutellarin and scutellarein induce apoptosis and trigger autophagy (Table 3) in cancer cells [52,143,144,145]. In a study conducted by Sun et al., scutellarin stimulated the phosphorylation of ERK1/2. Once phosphorylated and activated, it induced autophagy and apoptosis in PC-9 and H1975 cells [146]. Different studies show that combining scutellarin and scutellarein with commonly available anticancer drugs such as cisplatin enhances the sensitivity and efficacy of the commonly available anticancer compounds; in another study conducted by Sun et al., cisplatin, when combined with scutellarin, drastically inhibited the growth of cancer cells. Further, the study reported that scutellarin resulted in the activation of the p53 pathway mediated by ERK, which further resulted in caspase-3-mediated apoptosis. Further, the study reported that cisplatin-induced cytotoxic autophagy was enhanced by scutellarin [49,147,148,149,150]. Hou et al. demonstrated the role of scutellarin on the breast cancer cells MCF-7. The study demonstrated significant growth reduction in breast cancer cells treated with scutellarin. Additionally, it was found that scutellarin promoted apoptosis and autophagy by activating the HIPPO–YAP signaling pathway, supporting the potential therapeutic application of scutellarin-based drugs to provide patients with breast cancer the best possible outcomes [151,152]. The potential benefits of scutellarin and scutellarein need more clinical investigation for cancer treatment and as a newer therapeutic option [153,154,155,156,157].

## 6. Conclusions

In this review article, the molecular mechanisms of the flavone aglycones baicalein, scutellarein and wogonin and their glycosides baicalin and scutellarin derived from *Scutellaria* roots on the regulation of autophagy are discussed. Autophagy is known to play a dual-edged sword role in the carcinogenesis process, acting either as a protumorigenic or antitumorigenic mechanism, dependent on cancerous cell types and their degree of malignancy. Therefore, the studied flavones can behave as antiautophagic or proautophagic agents in suppressing tumorigenesis and malignant progression in diverse tumors. Therefore, understanding these fine cellular regulatory processes is important to better intervene in these mechanisms in the future, ultimately leading to safer and more efficient treatment modalities in the fight against different cancerous diseases.

## Figures and Tables

**Table 1:** An overview of the main features of wogonin, baicalein, baicalin, scutellarein and scutellarin.

Compound	Source Plant/Plants	Bioactivities	Reference
Wogonin	*Scutellaria baicalensis* Georgi radix, *Andrographis paniculata* Burm.f, *Anodendron affine* (Hook. & Arn.) Druce, *Tetracera indica* L.	Antioxidant, antiviral, anti-inflammatory, antiproliferative	[26,27,28]
Baicalein	*S baicalensis*, *S lateriflora*, *Oroxylum indicum*	Positive allosteric modulator of GABAA receptor, anxiolytic activity	[29,30,31,32]
Baicalin	*S. lateriflora*, *S. galericulata*, *Thalictrum baicalense*, *Radix scutellariae*	Neuroprotective, cardioprotective, antioxidant, antiatherosclerotic, antibacterial, anticoronaviral	[37,38]
Scutellarein	*S lateriflora*, *Scoparia dulcis*, *Artemisia douglasiana*, *Asplenium belangeri* (Fern)	Anticancer, antiproliferative, antioxidant	[41,42,43,52]
Scutellarin	*Scutellaria* spp., *Erigeron* spp.	Liver diseases, cerebrovascular diseases, hyperlipidemia	[46,51]

**Table 2:** Pharmacokinetic data of *Scutellaria* flavones.

Compound	Administration of Compound	Pharmacokinetic Analysis	Reference
**Wogonin (W.O.)**	Oral administration in C57BL/6 mice H9c2 cells	Modulated Gasdermin D protein in H9c2 cells; Attenuated CDDP-induced cardiotoxicity and showed antipyroptotic effects	[58]
	Oral administration in rats at 10, 20 and 40 mg/kg	Modulate the activities of CYPs, P-gp and C_max_ AUC_0-t_ of W.O. were proportionally increased	[59]
	Oral administration of R.S. extract (300 mg/kg) to Sprague–Dawley rats	W.O. showed the ability to cross the blood–brain barrier	[60]
	I.G. administration of W.O. in rats	Metabolized/detected in the small intestine and liver	[61]
	I.V. WO dose i.e., 10, 20 and 40 mg/kg I.G. 100 mg/kg dose I.V. WO (20 mg/kg) in Sprague–Dawley rats	W.O. was detected in all examined tissues; the highest levels were found in the kidney and liver, and 21% was excreted as an unchanged drug	[62]
**Baicalein**	Oral administration (121 mg/kg bw) and Pulmonary administration (10 mg/kg) in Male Sprague–Dawley (S.D.) rats	Oral baicalein nanocrystals: Bioavailability of baicalein is 1.67-fold, showing rapid and extensive absorption	[63]
	Oral administration Male SD rats 30 mg/kg baicalein	Distributed rapidly within 0.25 h and accumulated in the lung and liver Quickly absorbed in plasma lung > kidney >liver having T1/2z 8.08	[64]
	Oral administration in normal rats (65 mg/kg)	Baicalein was significantly higher in the stomach > liver > intestine	[65]
	In situ perfusion in Male Wistar rats	Baicalein was moderately absorbed as per the stomach > small intestine >colon	[66]
**Baicalin**	I.V. administration (230–250 g) of 37 µmol/kg to Male Wistar rats Oral administration of 227 µmol/kg	T ½ = 0.12 ± 0.02 in I.V. administered rats in plasma. Plasma concentration of baicalin displayed a second peak over the 8 –12 h (i.v.)	[67]
	I.V and oral administration in Male Sprague–Dawley rats	Rapid absorption and simultaneous glucuronidation/sulfation. The absorption rate was slower and the Cmax was lower for oral baicalin compared with I.V baicalein	[68]
	I.G. and oral administration in rats at a dose of 160 mg/kg	Coadministration significantly upregulated the C_max_, AUC0-t, and AUC0-∞ of oral dose by 2.02, 1.65, 1.66-fold in male rats	[69]
	Oral Administration to Male rats	Baicalein is significantly hydrolyzed in the gastrointestinal tract. The total cumulative amounts of baicalin were 54% of the doses	[70]
	Intestinal perfusion model: In rat in situ single-pass	In the rat’s intestinal regions, baicalin underwent considerable metabolism via conjugative processes	[71]
**Scutellarin/** **Scutellarein**	Oral administration 200 mg kg/bw in Male Wistar rats	Scutellarein is obtained after metabolizing scutellarin in the blood by glucuronic acid and methylating enzymes in the liver	[72]
	Oral administration of 80 mg kg/bw to Male Wistar rats	Two metabolites of Scutellarin, viz., Scutellarein 6,7-di-*o*-β-_D_-glucuronide and Scutellarein observed in urine	[73]
	I.V administration of 36 mg kg/bw to rats	Total of four metabolites were observed in the plasma. Scutellarin was metabolized via dehydroxylation and methylation	[74]
	Oral administration in human subject	Glucuronidation of scutellarin is mediated by uridine 5’-diphosphoglucuronosyltransferase (UGT) in rats and humans	[75]
	Scutellarin (S-7-G) at a dose of 75 mg/kg orally to Male Sprague–Dawley rats	S-7-G and S-6-G were spotted in the systemic circulation S-7-G absorbed as aglycone after hydrolyzed in the intestinal Glucuronidation of S-7-G occurs in liver microsomes of rat	[76]

**Abbreviations**: IV: Intravenous; IG: Intragasteric; CDP: cresyl diphenyl phosphate; µmol: micro mole; S7G: Scutellarin; SD: Sprague–Dawley; UGT: uridine 5’-diphosphoglucuronosyltransferase; CYPs: Cytochrome P450s.

**Table 3:** Regulation of autophagic machinery by *Scutellaria* flavones in different cancer types.

Scutellaria Flavones	Type of Cancer	Cell Line	Mechanisms of Action	Ref.
Wogonin	Pancreatic cancer	Panc-1 and Colo-357	↑ROS, Beclin-1/PI3K	[119]
Wogonin and oxaliplatin	Gastric cancer	BGC-823 cells	↑phospho-JNK (Thr183/Tyr185), phospho-ULK1 (Ser555), ↑LC3II	[120]
Wogonin	Nasopharyngeal cancer	NPC-TW076 and NPCTW039	↑LC3 I/II cleavage, autophagosome/autolysosome, ↓Raf/ERK	[158]
Wogonin	Colorectal cancer	SW1417, SW48, DLD-1, HCT-15, LS-180 and CCD-18Co	↓AKT and STAT3, ↑Beclin-1 caspases 3/8/9 and Bax expressions	[12]
Wogonin derivative GL-V9	Cutaneous squamous cancer	A431 cells	↓Akt/mTOR pathway	[118]
Baicalein	Breast cancer	MCF-7 and MDA-MB-231	↓PI3K/AKT, NF-κB	[159]
Baicalein	Glioma cells	U251 cells	↑LC3, ↑caspase-3, ↑phosphorylation of AMPK	[160]
Baicalein	Lung cancer	A549 and H1299	↑activated A.M.P.K., ↑Drp1-mediated mitochondrial fission	[135]
Baicalein	Ovarian cancer	HEY and A2780	↑LC3-II, ↑PARP, ↑phosphorylation of ERK, ↑Beclin-1	[133]
Baicalein	Hepatocellular cancer	SMMC-7721 and Bel-7402	↑endoplasmic reticulum (E.R.) stress, ↓Bcl-2, ↑J.N.K.	[153]
Baicalein	Thyroid cancer	F.R.O.	↓Bcl-2/Bax, ↑Caspase-3, ↑Caspase-8, ↑Beclin-1, Atg5, ↓ERK, ↓PI3K/Akt	[136]
Baicalein	Gastric cancer	MGC-803	↑LC3, ↓PI3K and ↓AKT, ↑P62	[161]
Baicalein	Prostate and breast cancer cell	PC-3, MDA-MB-231 and DU145	↓mTOR, ↑ activated AMPK/ULK1	[132]
Baicalein and cisplatin	Gastric cancer	SGC-7901 and SGC-7901/DDP	↓Akt/mTOR and Nrf2/Keap 1	[139]
Baicalein	Thyroid cancer	MDA-T68	↑Bax, ↓NF-kB, ↓Cyclin B1	[162]
Baicalin	Bladder cancer	T24 cells	↑Atg 5, ↑Atg 7, ↑Atg 12, ↑Beclin-1, ↑LC3-II	[163]
Baicalin	Osteosarcoma	HOS, MG63 U2OS and 143B	↓PI3K/Akt/mTOR, ↓ERK1/2, ↓β-catenin, ↑Bax, ↑caspase-3, ↑cleaved PARP	[140]
Baicalin	Hepatocellular cancer	SMMC-7721	↓CD147, ↑Beclin-1	[164]
Scutellarin	Breast cancer	MCF-7	↑p-YAP, ↓Y.A.P., ↑autophagy	[151]
Scutellarin	Lung cancer	PC-9 and H1975, HepG2, Hela	↑LC3-II, ↓p-AKT	[146]
Scutellarin and cisplatin	Lung adenocarcinoma	A549, PC-9, H1975, and A549/DDP	↑p53 and ↓c-met/AKT	[165]

## Data Availability

Not applicable.
